# Yeast Starter as a Biotechnological Tool for Reducing Copper Content in Wine

**DOI:** 10.3389/fmicb.2017.02632

**Published:** 2018-01-10

**Authors:** Angela Capece, Rossana Romaniello, Laura Scrano, Gabriella Siesto, Patrizia Romano

**Affiliations:** ^1^Scuola di Scienze Agrarie, Forestali, Alimentari ed Ambientali (SAFE), Università degli Studi della Basilicata, Potenza, Italy; ^2^Dipartimento delle Culture Europee e del Mediterraneo, Università degli Studi della Basilicata, Matera, Italy

**Keywords:** copper resistance, copper-reducing yeasts, wine, *Saccharomyces cerevisiae* biodiversity, biotechnological tools

## Abstract

Copper is widely used in agriculture as a traditional fungicide in organic farming to control downy mildew on grapes, consequently it is possible to find this metal during all stages of the vinification process. Low amounts of copper play a key role on the function of key cell enzymes, whereas excess quantities can exert amount-dependent cytotoxicity, resulting in general cellular damage. Nowadays the excessive copper ions in wines is removed by addition of adsorbents, but these additives can influence the sensory characteristics of wine, as well as detrimental to the health of consumers. It is well known that high concentrations of Cu^2+^ can be toxic to yeasts, inhibiting growth and activity, causing sluggish fermentation and reducing alcohol production. In this study, 47 *S. cerevisiae* strains were tested for copper tolerance by two different tests, growth on copper added medium and fermentative activity in copper added grape must. The results obtained by the two different tests were comparable and the high strain variability found was used to select four wild strains, possessing this characteristic at the highest (PP1-13 and A20) and the lowest level (MPR2-24 and A13). The selected strains were tested in synthetic and natural grape must fermentation for ability to reduce copper content in wine. The determination of copper content in wines and yeast cells revealed that at the lowest copper residual in wine corresponded the highest content in yeast cells, indicating a strong strain ability to reduce the copper content in wine. This effect was inversely correlated with strain copper resistance and the most powerful strain in copper reduction was the most sensitive strain, MPR2-24. This wild strain was finally tested as starter culture in cellar pilot scale fermentation in comparison to a commercial starter, confirming the behavior exhibited at lab scale. The use of this wild strain to complete the alcoholic fermentation and remove the copper from wine represents a biotechnological sustainable approach, as alternative to the chemical-physical methods, ensuring at the same time a completed alcoholic fermentation and organoleptic quality of wine.

## Introduction

In organic viticulture the control of downy mildew on grapes is based almost exclusively on copper, which is allowed to be used because considered a traditional fungicide in organic farming. The long-term use of copper led to an increased copper level not only in soil (Provenzano et al., 2010; Ash et al., 2012), but also in grape and must; copper salt addition for eliminating H_2_S (García-Esparza et al., 2006; Tamasi et al., 2010) may also increase the copper content in must and consequently in wine.

In biological vineyards the increased intake of copper compounds has caused high levels of copper residues on the grapes (Brandolini et al., 2002). In winemaking, elevated concentrations of this metal can be toxic to yeasts, affecting cell growth and activity; high level in must of Cu^2+^, such as 0.1 mM (Ohsumi et al., 1988) influences negatively yeast growth, inducing sluggish fermentation (Azenha et al., 2000) and a reduction in alcohol production (Mrvcić et al., 2007).

Moreover, the copper can influence wine strains activity in different ways: prevention or limiting of *Saccharomyces cerevisiae* growth, reduction of absorption of reducing sugars, which consequently causes a decrease on ethanol production. These effects were directly correlated with copper concentration and strain biodiversity (Sun et al., 2015). In *Saccharomyces cerevisiae*, the strains exhibit a wide variability in the level of copper tolerance (Capece et al., 2016) and in the cell capability to adsorb copper ions (Benítez et al., 2002; Mira et al., 2007; Schubert and Glomb, 2010). The adsorption of heavy metal in yeasts can be achieved by two ways, non-biological (dead cells) or biological (living cells) adsorption. Different studies reporting data on yeast biological adsorption of heavy metals are available, mainly addressed to the study of factors influencing the properties of heavy metal adsorption or the dynamic models of adsorption (Vasudevan et al., 2002, 2003). Furthermore, the adsorption by living cells can be subdivided as extracellular and intracellular adsorption (Chen et al., 2014). However, most of these results were related to industrial wastewater treatment systems, whereas few data on wine fermentation process are available. The studies on wine fermentation were based mainly on distinction between adsorption by dead or living cells, whereas Sun et al. (2016) reported results regarding extracellular or intracellular copper adsorption by living yeast cells. In this pathway, *S. cerevisiae* cells might firstly adsorb copper on cell surface, after the copper ions are moved into intracellular spaces.

In the first step, named as “passive biosorption” or extracellular, the interactions between metal-functional groups present on cell surface, such as carboxyl, phosphate, hydroxyl, amino, sulfur compounds, etc., capture metal ions to the cell surface. This process is independent from the metabolism, it starts very quickly (within several min) and it is a dynamic equilibrium of reversible adsorption–desorption, as the metal ions adsorbed on cell surface can be removed by different agents, such as other ions, chelating agent or acids.

During the second step, named as “active biosorption” or intracellular, metal ions enter in the cells by going through the cell membrane and it was an ongoing slow process.

It was recently reported that after copper adsorption, the cell surface and intracellular compartments of *S. cerevisiae* changed irregularly. A yeast strain copper resistant and able to accumulate this metal in the cell was patented with aim to clean copper from extracellular solutions (Abe and Horikoshi, 2001). Recent results (Sun et al., 2015) demonstrated that in *S. cerevisiae* the principal mechanism involved in copper adsorption during alcoholic fermentation was cell surface adsorption, which reaches saturation in 24 h.

Due to detrimental effects in high concentrations, “maximum residue levels” (MSL) of copper in European and South African regulations have been established in 20 mg L^−1^ in grape must and 1 mg L^−1^ in wine (García-Esparza et al., 2006). Nowadays the excessive copper ions in wines is removed by addition of adsorbent such as glue; recently OIV allowed to add some additives, such as potassium ferrocyanide, bentonite, gum Arabic, polyvinylimidazole, polyvinylpyrrolidone copolymers, chitin, chitosan etc., but these treated wines have a lower content of polyphenols and aromatic compounds, which is reflected in the organoleptic properties of wine (Benítez et al., 2002). Anyway copper is unavoidable in winemaking and the adverse effects of long-term copper fungicide use can be just diminished by reducing the number of applications and doses of conventional copper fungicides and by combining this strategy with increasing use of biological preparations.

In this work, the variability for copper adsorption among wild *S. cerevisiae* strains allowed to select strains able to reduce excessive copper content in wine. The aim was to promote the utilization of a biotechnological method, alternative to chemical removal, ensuring at the same time a completed alcoholic fermentation and organoleptic quality of wine.

## Materials and methods

### Yeast strains

In this study 47 *S. cerevisiae* strains were used (Table 1): 44, belonging to the collection of the University of Basilicata, were isolated during spontaneous fermentation of grapes sampled in different areas and previously characterized for enological parameters, and three are commercial starters. Yeast cells were maintained on slants in YPD medium (1% w/v yeast extract, 2 w/v% bacto peptone, 2 w/v% glucose, 2 w/v% agar) at 4°C.

**Table 1:** *Saccharomyces cerevisiae* strains used in this study.

**Strain**	**Origin**	**References**
ND-14; CD2-6SC2; ND7; RB3-7SC2; TA8-4SC2; CB1-7SR3	Nero d'Avola variety, Sicily region	Capece et al., 2010
5TB8-60	Bosco variety, Liguria region	Capece et al., 2012
M1-47; M3-60; M3-59; M3-80;	Aglianico variety, Basilicata region	Capece et al., 2014
B7; A13; A14; A20; A21; 10_1_; 10_2_; B51	Aglianico del Vulture variety, Basilicata region	This study^*^
4LB; AGME	Aglianico del Vulture variety, Basilicata region	Capece et al., 2011b
PP1-1; PP1-15; PP1-31; PP2-22; PP1-13; MPR2-18; MPR2-42;	Primitivo variety, Basilicata region	This study^*^
MPR2-43; MPR2-28; MPR2-24; MPR2-26; BP1-29; BP2-17; BP2-33; BP1-13; BP1-33		
SC2-37; SB5-15; SB5-18; SA7-13	Sangiovese variety, Tuscany region	Capece et al., 2013
BA-215	Sangiovese variety, Tuscany region	Capece et al., 2011b
SN41	Sangiovese variety, Tuscany region	Brandolini et al., 2002
TA4-10	Inzolia variety, Sicily region	Capece et al., 2011a
EC1118	Commercial strain	Lallemand
796 AWRI	Commercial strain	Maurivin
FI5	Commercial strain	Laffort
ES 454	Commercial strain	Enartis

* *These strains were characterized in this study*.

### Strain resistance to copper

The strain resistance to copper was assessed both by evaluating the influence of copper, added as copper sulfate (CuSO_4_), on growth and fermentative activity of strains.

The copper influence on strain growth was tested by inoculating approximately 1 × 10^6^ cells/ml on solid synthetic medium, containing 6.7 g L^−1^ YNB (Yeast Nitrogen Base without amino acids and sulfate), 20 g L^−1^ glucose, added with increasing levels of CuSO_4_ (50, 100, 200, 300, 400, and 500 μmol L^−1^), in comparison to the control (the same medium without copper addition). After incubation at 26°C for 24 h, the strain resistance level to copper was defined as the lowest concentration of the metal allowing strain growth.

To evaluate the effect of copper on fermentative activity, each strain was inoculated (10^7^ cell/mL from pre-cultures grown for 24 h in YPD) in 10 mL of pasteurized grape must (100°C for 20 min), supplemented with 300 mg L^−1^ of CuSO_4_. As control, pasteurized grape must without copper addition was used. The copper resistance (FVR) was expressed as ratio between strain fermentative vigor in copper-added fermentations (Cu-FV) and the fermentative vigor without Cu addition (C-FV).The fermentative vigor was measured as the amount of CO_2_ produced at the third day of fermentation.

### Strain ability to reduce copper content in synthetic wine

On the basis of previous results, four wild strains were selected (MPR2-24, A13, PP1-13, A20) and tested in fermentation of synthetic grape must (SGM) in order to evaluate the strain ability to reduce the copper content in winemaking. As SGM, the medium reported by Henschke and Jiranek (1993) was used. Fermentations were conducted at 26°C in 130-mL Erlenmeyer flasks, equipped with Müller valves containing sulphuric acid and filled with 100 ml of SGM. The synthetic must was added with 300 μmol L^−1^ of CuSO_4_; as control, SGM without copper addition was used. The SGM was inoculated with 10^7^ cells mL-1, from a pre-culture grown in SGM for 24 h, and the fermentations were daily monitored by analyzing the weight loss. All the experiments were performed in triplicate. At the end of the process (when weight loss was less than 0.02 g for 2 days), the samples were centrifuged at 4.000 rpm for 10 min at 4°C. Both the obtained fractions (fermented samples and yeast cells) were stored at −20°C until required for analysis.

For copper determination in synthetic and natural wines, the samples, previously filtered through a 0.45 μm membrane filter, were degassed using an ultrasonic bath, while the yeast cells were submitted to the acid digestion prior filtration and analysis. Successively, each sample was added with HNO_3_ solution and mixed with internal standard (2 ppm Yttrium) by means of a fitting (T) positioned after the peristaltic pump. The copper level was determined according to EPA 6020A. Standard was purchased from Sigma-Aldrich (USA) and all analytical solvents used during the analysis were furnished from Levanchimica (Bari, Italy). The copper quantification in the alcohol matrix was carried out using an ICP-MS ICAP TM 7400 of Thermo Scientific (USA), equipped with an automatic sampler. The operating conditions used were: power 1.2 kW, gas flow 15.0 L/min, gas flow 2.25 L/min, spraying pressure 220 kPa, pump speed 18 rpm, wavelength of Cu 327.395 nm. Three replications were performed on each sample.

The strain ability to reduce copper content in synthetic wine (RCuSW) was calculated on the basis of the following equation: RCuSW = CuSW-CuSC, where CuSW and CuSC are copper content in copper added and control synthetic wine, respectively. The copper adsorption by strain (AsCuY) was calculated on the basis of the following equation: AsCuY = YCuSW-YCuSC, where YCuSW and YCuSC were copper content in yeast cells from copper added and control synthetic wine, respectively.

### Strain ability to reduce copper content in wine

The four wild selected strains were tested in inoculated fermentation at laboratory scale in pasteurized natural grape must (NGM). The NGM used was “Aglianico del Vulture,” presenting the following characteristics: pH 3.7; total soluble solids 227 g L^−1^; yeast assimilable nitrogen 281 mg L^−1^. The fermentations were performed following the protocol previously reported for SGM. The experimental wines and yeast cells recovered at the end of the process were analyzed for copper content, by using the protocol previously described. The strain ability to reduce copper content in wine (RCuW) was calculated on the basis of the following equation: RCuW = CuW-CuC, where CuW and CuC are copper content in copper-added and control wine, respectively. The copper adsorption by strain (AwCuY) was calculated on the basis of the following equation: AwCuY = YCuW-YCuC, where YCuW and YCuC were copper content in yeast cells from copper-added and control wine, respectively.

### Analytical profiles of experimental wines

Experimental wines obtained from NGM fermentation were analyzed for conventional chemical parameters, such as total and volatile and total acidity, residual sugars, alcohol, were measured using Fourier Transfer Infrared WineScan (FOSS, Hillerød, Denmark). The content of the main secondary compounds influencing wine aroma, such as higher alcohols (*n*-propanol, isobutanol, amyl alcohols), acetaldehyde, ethyl acetate, were determined by direct injection gas chromatography, whereas other volatile compounds, such us esters, volatile fatty acids, alcohols, aldehydes, ketones, were analyzed by SPME-GC-MS, following the methods described by Capece et al. (2013).

### Pilot scale fermentations

Pilot scale fermentations were performed by using the selected indigenous starter (MPR2-24) in comparison to the commercial strain ES 454 (ENARTIS), commonly used by the producer. The trials were performed in a cellar using grapes from vineyard following organic farming system. The fermentations were performed in sulphited (50 mg L^−1^) grape must (240 g L^−1^ sugar, pH 3.5) in 100 L stainless steel and inoculated with 1 × 10^7^ cells ml^−1^. The fermentation processes were daily monitored by determining sugar consumption. The final wines were analyzed for content of secondary compounds, conventional chemical parameters and the copper content, following the protocols previously reported. The copper removal ratio (CuRR) was calculated following this equation: CuRR = CuM-CuW/CuM, where CuM is copper concentration in grape must and CuW is copper level in wine. The implantation ability of each starter was evaluated by yeast isolation on WL medium (Pallmann et al., 2001) from wine samples, collected at the end of the process from each fermentation vessel; a representative number of yeast colonies (at least 20), randomly chosen from each sample, were submitted to amplification of inter-delta region, in comparison to inoculated starters. The starter implantation level was calculated as previously reported (Capece et al., 2012).

### Data analysis

Statistical software (PAST software ver. 1.90; Hammer et al., 2001) was used for analyzing all data. Data of volatile compounds and copper content in wines and yeast cells were analyzed using one-way Analysis of Variance (ANOVA) to compare the mean values between fermentations with and without copper addition.

## Results

### Evaluation of strain resistance to copper

Forty-seven *S. cerevisiae*, 44 wild and three commercial strains, were tested for copper sensitivity, in particular for tolerance to copper sulfate, the copper formulation applied as a fungicide to treat powdery mildew in vineyards. The evaluation of copper influence on yeast growth revealed a wide strain variability on YNB medium. In particular, about 60% of the strains exhibited low copper tolerance (the maximum tolerated doses were 100–200 μmol L^−1^), 15% of the strains were high copper tolerant (growing on the maximum tested dose), whereas the remaining strains grew at concentrations ranging between 300 and 400 μmol L^−1^ of CuSO_4_.

**Figure 1:**
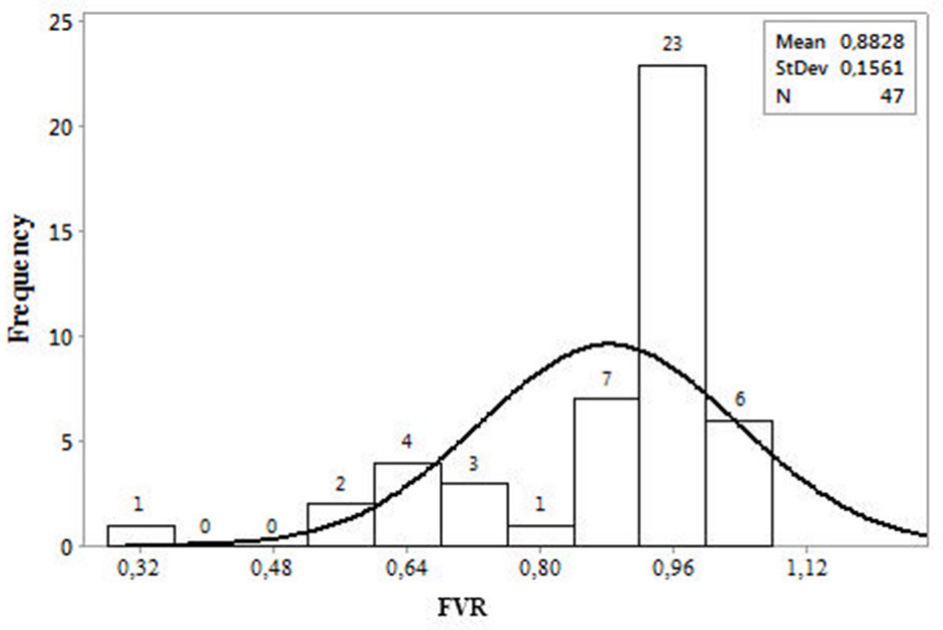
Distribution of 47 strains in function of copper resistance (FVR). FVR is expressed as ratio between strain fermentative vigor in copper-added fermentations (Cu-FV) and the fermentative vigor without Cu addition (C-FV). The fermentative vigor was measured as the amount of CO_2_ produced at the third day of fermentation.

The copper influence on strain fermentative activity was evaluated as strain ability to tolerate this compound, preserving its fermentative performance. As reported in Figure 1 and Table S1, six strains resulted very high copper tolerant, as the fermentative vigor was not affected by the presence of the compound (FVR values equals to or higher than 1), numerous strains (23) were slightly affected by the copper addition (FVR was about 0.96), whereas few strains exhibited a very low copper tolerance as the fermentative vigor was reduced at about 50% (or more) by copper addition (FVR values ranging between 0.32 and of 0.64).

It has be underlined that the results obtained by evaluating copper influence on fermentative activity confirmed those obtained by testing the copper effect on strain growth in synthetic medium; in fact, in both the tests, the most sensitive strain was MPR2-24.

On the basis of these results, four wild *S. cerevisiae* strains, exhibiting the lowest (MPR2-24, A13) and the highest copper sensitivity (PP1-13, A20) were selected for further characterization.

### Evaluation of strain ability to reduce copper content in synthetic wine

**Figure 2:**
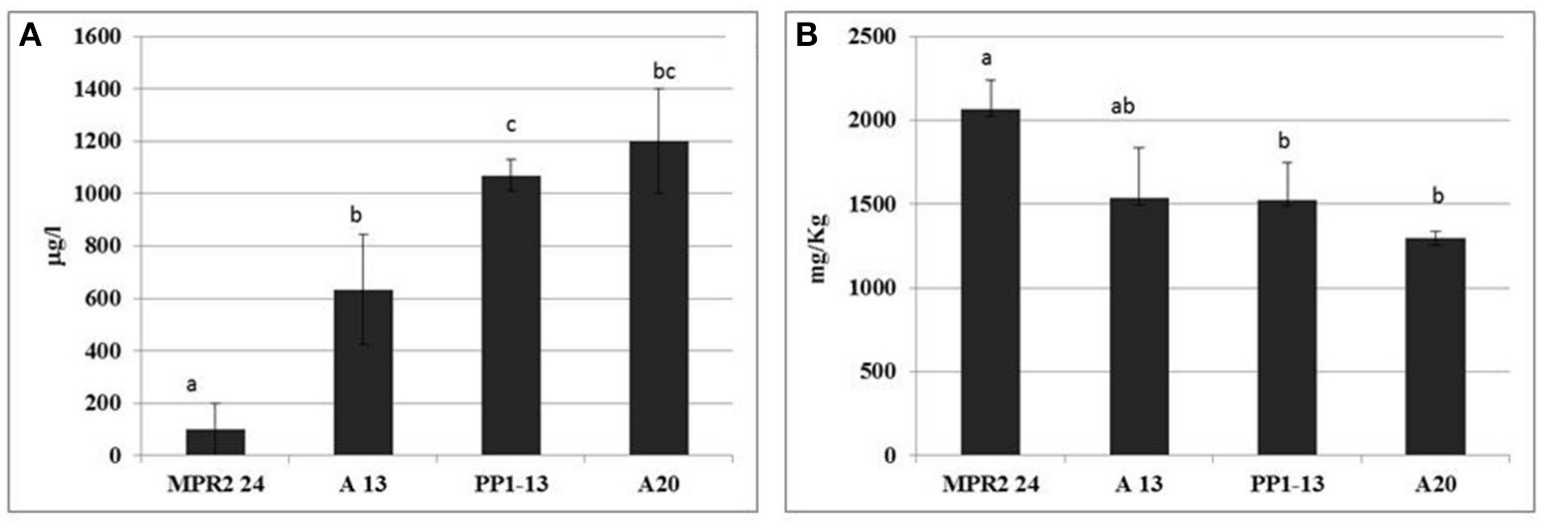
Strain ability to reduce copper content in synthetic grape must (SGM) fermentation. **(A)** Residual copper content in synthetic wine (RCuSW), calculated on the basis of the following equation: RCuSW = CuSW-CuSC, where CuSW and CuSC are copper content in copper added and control synthetic wine. **(B)** Copper adsorption by strain (AsCuY), calculated on the basis of the following equation: AsCuY = YCuSW-YCuSC, where YCuSW and YCuSC were copper content in yeast cells from copper added and control synthetic wine. Data are means ± SD of three independent experiments; different superscript letters indicate significantly different values (one-way ANOVA, *p* < 0.05).

The four selected strains were tested in SGM fermentation added with CuSO_4_, in comparison to the control, in order to test the strain ability to reduce copper content of wine. The monitoring of fermentative process revealed that all the fermentations (copper-added and controls) were completed, although the copper affected significantly the fermentative performance of sensitive strains and the duration of fermentation process (data not shown). Samples obtained at the end of the fermentations (synthetic wines and yeast cells recovered after centrifugation) were analyzed for copper content. The strain influence on copper content of synthetic wine is reported in Figure 2A. MPR2-24 exhibited the highest strain ability to reduce copper content in synthetic wine (RCuSW); in fact the lowest copper residual was detected in synthetic wine fermented by this strain, which was significantly different from all the other samples. On the contrary, the lowest RCuSW was shown by the two resistant strains, PP1-13 and A20, which determined the highest copper residual in the samples.

The determination of copper residual in yeast cells (Figure 2B) showed that the highest residual content was detected in the cell pellet of the sensitive strain MPR2-24, which contained about 2.050 mg kg^−1^, whereas the copper residual content detected in cells of the other strains ranged between 1.300 and 1.530 mg kg^−1^. As a consequence, MPR2-24 resulted the strain with the highest RCuSW and AsCuY (copper adsorption by strain).

### Evaluation of strain ability to reduce copper content in natural wines

**Figure 3:**
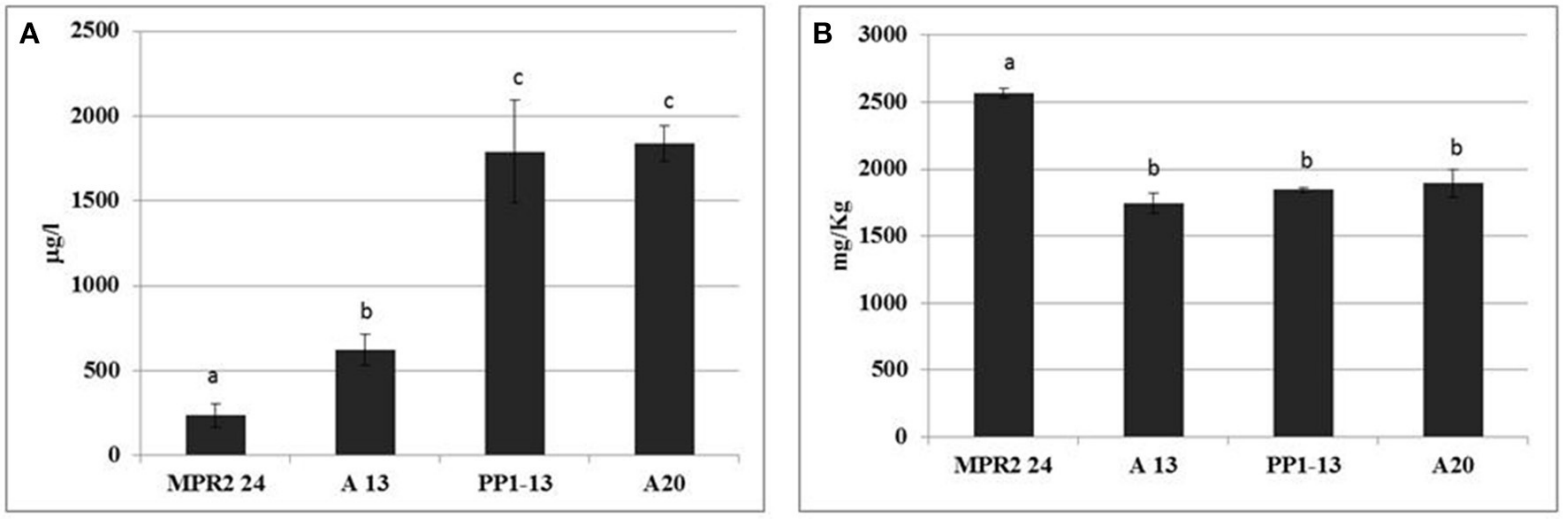
Strain ability to reduce content in natural grape must (NGM) fermentation. **(A)** Residual copper content in wine (RCuW), calculated on the basis of the following equation: RCuW = CuW-CuC, where CuW and CuC are copper content in copper added and control synthetic wine. **(B)** Copper adsorption by strain (AwCuY), calculated on the basis of the following equation: AwCuY = YCuW-YCuC, where YCuW and YCuC were copper content in yeast cells from copper-added and control wine, respectively. Data are means ± SD of three independent experiments; different superscript letters indicate significantly different values (one-way ANOVA, *p* < 0.05).

In order to confirm the strain ability to reduce copper content also in wines from natural grape must, the experimental wines and yeast cells (separated by centrifugation from final samples) were analyzed for copper content. The strain ability to reduce copper content in wines (RCuW) is reported in Figure 3A. A different behavior was found in function of strain copper sensitivity: the highest reduction level was obtained by the sensitive strain, MPR2-24, followed by the other copper sensitive strain A13, whereas the highest copper content was detected in wines produced by the two resistant strains (PP1-13 and A20), which exhibited a behavior very similar, with a copper content ranging between 1.790 and 1.830 mg L^−1^. The residual copper content adsorption by yeast cells (AwCuY) recovered at the end of the fermentative process (Figure 3B) revealed that the highest level was detected in MPR2-24 cells (about 2.600 mg kg^−1^), with a significantly higher level than those found in the other strain cells (values ranging between about 1.750 and 1.900 mg kg^−1^).

It has be underlined that the lowest level of copper was found in wine obtained by inoculating MPR2-24 and the highest copper content was detected in yeast cells of the same strain, confirming the results obtained in SGM fermentations. These results outline the potential ability of MPR2-24 strain to remove copper content from wine.

### Copper influence on fermentative performance of selected strains in NGM

The evolution of fermentative process and chemical parameters detected in the experimental wines from NGM are shown in Table 2. All the data related to strain fermentative performance, such as fermentative vigor (FV) and power (FP), reflected the different copper sensitivity of the strains. In fact, statistically significant differences between values detected in fermentation with and without CuSO_4_ addition were found for copper sensitive strains (MPR2-24 and A13). For these strains, a low fermentation activity was found in copper-added must, with a FP decrease of 23% (MPR2-24) and 35% (A13) and, consequently, high residual sugars in final wines were detected in fermentation with CuSO_4_ addition than values detected in the control (Table 2). No influence of CuSO_4_ supplementation on strain FV and FP was found for copper tolerant strains (PP1-13, A20). However, all the strains completed the fermentation (1.23 g L^−1^ maximal residual sugars), although the processes were delayed for sensitive strains in grape must containing copper.

**Table 2:** Main technological characteristics of selected *S. cerevisiae* strains.

**Strain**	**FT**	**FV**	**FP**	**Residual sugars gL^−1^**	**Total acidity gL^−1^**	**Volatile acidity gL^−1^**	**Ethanol % v/v**
MPR2-24	C	1.4 ± 0.21^*^	0.81 ± 0.05^*^	0.43 ± 0.12^*^	8.17 ± 0.15	0.30 ± 0.09^*^	9.39 ± 0.07^*^
	Cu	0.35 ± 0.25	0.63 ± 0.02	0.87 ± 0.06	8.44 ± 0.15	0.79 ± 0.03	9.06 ± 0.09
A13	C	1.63 ± 0.03^*^	1.11 ± 0.01^*^	0.83 ± 0.06^*^	8.68 ± 0.69	0.05 ± 0.08^*^	9.80 ± 0.02^*^
	Cu	0.53 ± 0.29	0.71 ± 0.15	1.23 ± 0.06	8.40 ± 0.07	0.28 ± 0.02	8.45 ± 0.47
PP1-13	C	1.45 ± 0.11	1.11 ± 0.03	0.57 ± 0.06	9.66 ± 0.14	0.59 ± 0.08	9.84 ± 0.10^*^
	Cu	1.53 ± 0.11	1.11 ± 0.05	0.60 ± 0.10	9.68 ± 0.14	0.65 ± 0.08	9.23 ± 0.14
A20	C	1.53 ± 0.08	1.05 ± 0.02	0.57 ± 0.12	9.53 ± 0.12	0.66 ± 0.02	9.54 ± 0.18
	Cu	1.50 ± 0.08	1.13 ± 0.10	0.40 ± 0.17	9.58 ± 0.36	0.48 ± 0.17	9.40 ± 0.06

*FT, fermentation type; C, fermentation in grape must (control); Cu, fermentation in Cu-added grape must*.

*FV, strain fermentative vigor expressed as g CO_2_/day measured at the second fermentation day*.

*FP, strain fermentative power expressed as g CO_2_/day measured at the end of the fermentation*.

*Data are mean ± SD of three independent experiments. For each strain, the asterisk indicates significantly different values (one-way ANOVA, P < 0.05) between wines from control and Cu-added grape must*.

Otherwise for all the strains, no significant differences between the two fermentations were found in the levels of total acidity, while the ethanol content (ranging between 8.45 and 9.84% v/v) was significantly higher in wines obtained from fermentation without CuSO_4_ for all the strains, except for A20. In wines obtained by the two sensitive strains, the copper supplementation affected significantly the volatile acidity, determining a considerable increase.

### Copper influence on analytical profiles of wines produced by selected strains

The experimental wines obtained from the two fermentations were analyzed for content of by-products related to wine aroma, in order to evaluate the influence of copper on strain metabolic behavior. Among the compounds detected by gas-chromatography (Table 3), acetaldehyde was produced in the highest amounts in copper added fermentation (except for MPR2-24 strain), although statistically significant differences were found only for wines obtained by inoculating PP1-13 strain. The production levels of isobutanol, *n*-propanol and amyl alcohols were significantly affected by copper addition in fermentations with sensitive strains, mainly for A13, which produced a lower level of these by-products in wines obtained from copper-added must. Also the ethyl acetate production was significantly affected by copper addition for sensitive strains, although in different way in the two strains. The analysis of the volatile fraction by SPME-GC-MS of the experimental wines allowed the identification of 49 compounds, belonging to different chemical classes, such as esters, alcohols, aldehydes (Table 4). Among the esters, the compounds present in the highest amounts were ethyl propanoate, ethyl isobutyrate, isobutyl acetate, ethyl butyrate, isoamyl acetate, ethyl hexanoate, 2-phenylethyl acetate, whereas 1-hexanol, benzyl alcohol, 2-phenylethanol were the alcohols present in the highest concentration and furfural was the main aldehyde. By analyzing the influence of copper addition on production level of these compounds, no statistically significant differences were found in wines obtained with and without copper addition for the resistant strains A20 and PP1-13 (wines from PP1-13 significantly differed only for cis-3-Hexen-1-ol content) and for sensitive strain MPR2-24. On the contrary, wines obtained inoculating A13 strain with copper addition differed significantly from the control for numerous volatile compounds, such as ethyl butyrate, isoamyl acetate, ethyl valerate, isoamyl butyrate, methyl octanoate, ethyl 6-hydroxyhexanoate, 1-pentanol, 2-heptanol, benzyl alcohol, 2-phenylethanol, linalool, β-citronellol.

**Table 3:** By-products (mg L^−1^) in experimental wines produced by the four *S. cerevisiae* strains fermentation with and without copper addition.

	**MPR2-24**		**A13**		**PP1–13**		**A20**	
	**C**	**Cu**	**C**	**Cu**	**C**	**Cu**	**C**	**Cu**
Acetaldehyde	34.88 ± 4.93	34.46 ± 2.46	48.61 ± 3.70	53.42 ± 2.76	33.71 ± 3.21^*^	50.50 ± 4.01	35.86 ± 2.29	41.66 ± 6.50
Ethyl acetate	14.67 ± 0.15^*^	20.89 ± 3.09	18.16 ± 1.01^*^	14.11 ± 1.06	26.23 ± 0.95	25.64 ± 2.26	27.54 ± 1.84	30.81 ± 4.57
*n*-Propanol	29.17 ± 0.87	39.51 ± 6.42	67.13 ± 2.86^*^	48.04 ± 2.45	52.19 ± 3.15	65.55 ± 0.63	69.13 ± 3.99^*^	112.52 ± 10.21
Isobutanol	48.19 ± 1.20^*^	34.45 ± 2.93	46.36 ± 3.09^*^	39.63 ± 2.98	54.89 ± 4.62	55.41 ± 2.65	41.12 ± 4.80	40.41 ± 1.31
Amyl alcohols	184.26 ± 0.42^*^	126.64 ± 1.90	205.64 ± 27^*^	148.48 ± 4.34	159.14 ± 2.14	160.97 ± 2.65	155.82 ± 4.80	164.28 ± 1.31

*C, fermentation in grape must (control). Cu, fermentation in Cu-added grape must. Data are mean ± SD of three independent experiments For each strain, the asterisk indicates significantly different values (one-way ANOVA, P < 0.05) between wines from control and Cu-added grape must*.

**Table 4:** Volatile compounds (μg L^−1^) in experimental wines produced by the four *S. cerevisiae* strains in fermentation with and without copper addition.

**Compounds**	**MPR2-24**		**A13**		**PP1–13**		**A20**	
	**C**	**Cu**	**C**	**Cu**	**C**	**Cu**	**C**	**Cu**
**ESTERS**								
Ethylpropanoate	122.84 ± 13.2	102.44 ± 6.68	90.39 ± 14.44	119.97 ± 22.4	137.89 ± 2.91	139.68 ± 39.93	140.10 ± 18.41	153.03 ± 5.16
Ethylisobutyrate	189.98 ± 19.86	156.64 ± 10.21	130.73 ± 28.16	167.75 ± 7.84	210.35 ± 5.15	242.51 ± 31.48	214.18 ± 28.14	233.17 ± 8.25
Ethylbutanoate	1.23 ± 0.28	1.11 ± 0.37	0.99 ± 0.08	0.97 ± 0.30	1.08 ± 0.23	1.14 ± 0.46	1.22 ± 0.16	1.15 ± 0.33
Propyl acetate	47.03 ± 5.83	38.14 ± 2.90	32.59 ± 5.38	43.55 ± 7.94	50.34 ± 0.61	50.69 ± 14.38	50.78 ± 6.67	55.44 ± 1.88
Isobutyl acetate	96.56 ± 21.91	70.15 ± 4.56	60.45 ± 10.99	82.56 ± 14.49	94.20 ± 2.16	96.05 ± 26.57	95.83 ± 12.59	104.69 ± 3.53
Ethylbutyrate	97.74 ± 22.18	70.73 ± 4.66	49.89 ± 20.62^*^	87.70 ± 10.54	96.32 ± 1.06	101.34 ± 21.18	97.01 ± 12.75	105.88 ± 3.60
Ethyl 2-methylbutanoate	5.65 ± 1.28	5.08 ± 1.70	4.32 ± 0.24	4.55 ± 1.26	4.87 ± 1.23	5.34 ± 1.99	5.61 ± 0.74	5.27 ± 0.82
Ethyl 3-methylbutanoate	1.95 ± 0.44	1.75 ± 0.59	1.76 ± 0.27	1.47 ± 0.59	1.79 ± 0.24	1.75 ± 0.84	1.94 ± 0.25	1.82 ± 0.53
Ethyl 2-methylpropanoate	2.00 ± 0.45	1.80 ± 0.60	1.38 ± 0.13	1.67 ± 0.36	1.66 ± 0.54	1.95 ± 0.62	1.99 ± 0.26	1.87 ± 0.54
Ethylisovalerate	0.92 ± 0.21	0.83 ± 0.28	0.33 ± 0.31	0.88 ± 0.11	0.65 ± 0.46	1.01 ± 0.15	0.92 ± 0.12	0.86 ± 0.25
Butyl acetate	1.35 ± 0.31	1.21 ± 0.41	0.45 ± 0.49	1.30 ± 0.17	0.93 ± 0.69	1.48 ± 0.21	1.34 ± 0.18	1.26 ± 0.37
Isoamyl acetate	501.89 ± 113.89	363.35 ± 23.88	218.00 ± 138.62^*^	464.27 ± 50.90	495.26 ± 5.27	479.28 ± 12.99	498.10 ± 65.45	545.62 ± 18.09
Ethylvalerate	1.02 ± 0.23	0.92 ± 0.31	0.42 ± 0.30^*^	0.95 ± 0.11	0.73 ± 0.47	1.09 ± 0.18	1.01 ± 0.13	0.95 ± 0.28
Methylhexanoate	1.64 ± 0.37	1.48 ± 0.50	0.65 ± 0.51	1.54 ± 0.18	1.18 ± 0.77	1.77 ± 0.28	1.63 ± 0.21	1.53 ± 0.45
Ethylhexanoate	103.02 ± 23.38	74.95 ± 4.87	74.49 ± 3.62	101.56 ± 15.79	101.46 ± 1.15	115.91 ± 15.01	102.25 ± 13.44	111.38 ± 3.90
Isoamylbutyrate	3.15 ± 0.71	2.83 ± 0.95	1.25 ± 0.97^*^	2.96 ± 0.34	2.25 ± 1.48	3.40 ± 0.54	3.12 ± 0.41	2.94 ± 0.85
Hexyl acetate	6.67 ± 1.51	6.00 ± 2.01	4.31 ± 0.65	7.58 ± 2.60	5.44 ± 1.99	8.51 ± 1.95	6.62 ± 0.87	6.22 ± 1.81
Ethylheptanoate	1.07 ± 0.24	0.96 ± 0.32	0.43 ± 0.33	1.00 ± 0.11	0.77 ± 0.50	1.15 ± 0.18	1.06 ± 0.14	1.00 ± 0.29
Ethyl trans-2-hexenoate	1.36 ± 0.31	1.22 ± 0.41	0.36 ± 0.57	1.34 ± 0.21	0.90 ± 0.76	1.53 ± 0.20	1.35 ± 0.18	1.27 ± 0.37
Isobutylhexanoate	0.16 ± 0.04	0.14 ± 0.05	0.14 ± 0.02	0.48 ± 0.57	0.15 ± 0.02	0.50 ± 0.55	0.16 ± 0.02	0.15 ± 0.04
Methyloctanoate	0.91 ± 0.21	0.82 ± 0.27	0.44 ± 0.21^*^	0.83 ± 0.09	0.69 ± 0.37	0.95 ± 0.19	0.90 ± 0.12	0.85 ± 0.25
Ethyloctanoate	8.75 ± 1.98	7.87 ± 2.64	6.54 ± 0.35	9.05 ± 1.96	7.48 ± 2.00	10.26 ± 1.44	8.68 ± 1.14	8.16 ± 2.37
Isoamylhexanoate	4.29 ± 0.97	3.86 ± 1.29	1.96 ± 1.11	3.94 ± 0.43	3.17 ± 1.84	4.54 ± 0.83	4.26 ± 0.56	4.00 ± 1.17
Ethylnonanoate	0.11 ± 0.02	0.10 ± 0.03	0.04 ± 0.03	0.11 ± 0.03	0.08 ± 0.05	0.13 ± 0.02	0.11 ± 0.01	0.10 ± 0.03
Methyldecanoate	0.13 ± 0.03	0.11 ± 0.04	0.10 ± 0.01	0.10 ± 0.03	0.11 ± 0.02	0.12 ± 0.05	0.13 ± 0.02	0.12 ± 0.03
Isoamyloctanoate	3.06 ± 0.70	2.76 ± 0.92	1.67 ± 0.55	2.71 ± 0.36	2.37 ± 1.13	3.14 ± 0.72	3.04 ± 0.40	2.86 ± 0.83
Ethylphenylacetate	7.89 ± 1.79	7.09 ± 2.38	4.47 ± 1.29	6.92 ± 0.98	6.18 ± 2.79	8.02 ± 1.92	7.83 ± 1.03	7.36 ± 2.14
2-Phenylethyl acetate	93.25 ± 21.16	67.88 ± 4.40	77.93 ± 7.99	96.36 ± 20.71	82.97 ± 15.75	109.34 ± 15.24	92.54 ± 12.16	100.95 ± 3.46
Ethyl 6-hydroxyhexanoate	0.88 ± 0.20	0.79 ± 0.27	0.25 ± 0.35^*^	0.91 ± 0.20	0.59 ± 0.48	1.04 ± 0.15	0.87 ± 0.11	0.82 ± 0.24
**ALCOHOLS**								
1-Pentanol	2.00 ± 0.45	1.80 ± 0.60	0.66 ± 0.74^*^	1.93 ± 0.25	1.38 ± 1.03	2.21 ± 0.31	1.99 ± 0.26	1.87 ± 0.54
4-Methyl-1-pentanol	1.24 ± 0.28	1.12 ± 0.37	0.85 ± 0.08	1.03 ± 0.22	1.03 ± 0.34	1.21 ± 0.38	1.23 ± 0.16	1.16 ± 0.34
2-Heptanol	0.83 ± 0.19	0.74 ± 0.25	0.41 ± 0.18^*^	0.75 ± 0.09	0.62 ± 0.33	0.86 ± 0.17	0.82 ± 0.11	0.77 ± 0.22
1-Hexanol	112.99 ± 25.64	81.45 ± 5.48	75.56 ± 9.05	97.93 ± 15.35	111.35 ± 1.22	113.70 ± 9.19	112.14 ± 14.73	105.36 ± 30.66
cis-3-Hexen-1-ol	0.01 ± 0.00	0.01 ± 0.00	0.04 ± 0.05	0.01 ± 0.01	96.89 ± 11.37^*^	33.46 ± 17.93	0.01 ± 0.00	0.01 ± 0.00
1-Octen-3-ol	0.31 ± 0.07	0.28 ± 0.09	0.34 ± 0.09	0.31 ± 0.06	0.31 ± 0.00	0.36 ± 0.05	0.31 ± 0.04	0.29 ± 0.08
1-Heptanol	0.17 ± 0.04	0.15 ± 0.05	0.09 ± 0.03	0.15 ± 0.02	0.13 ± 0.06	0.17 ± 0.04	0.17 ± 0.02	0.16 ± 0.05
Benzylalcohol	148.82 ± 33.77	107.16 ± 7.27	72.02 ± 34.76^*^	134.97 ± 15.34	111.67 ± 61.03	155.73 ± 30.46	158.14 ± 3.66	161.25 ± 5.47
2-Phenylethanol	414.82 ± 51.50	325.47 ± 21.83	267.63 ± 63.65^*^	422.01 ± 47.21	358.13 ± 151.28	439.80 ± 4.36	480.85 ± 10.71	490.57 ± 16.27
linalool	11.82 ± 2.68	10.63 ± 3.56	5.69 ± 2.79^*^	10.73 ± 1.22	11.61 ± 0.16	12.38 ± 2.41	11.73 ± 1.54	11.02 ± 3.21
trans-Linalooloxide	0.02 ± 0.00	0.02 ± 0.01	15.22 ± 0.82	8.23 ± 1.00	11.24 ± 1.10	6.44 ± 3.74	0.02 ± 0.00	0.02 ± 0.01
cis-Linalooloxide	0.00 ± 0.00	0.00 ± 0.00	7.56 ± 3.57	2.76 ± 1.78	3.00 ± 5.19	2.75 ± 4.77	0.00 ± 0.00	0.00 ± 0.00
a-Terpineol	13.02 ± 2.95	11.71 ± 3.93	10.37 ± 0.75	10.32 ± 3.14	11.38 ± 2.55	12.14 ± 4.83	12.92 ± 1.70	13.44 ± 1.33
b-Citronellol	10.72 ± 2.43	9.64 ± 3.23	4.92 ± 2.74^*^	9.82 ± 1.08	10.55 ± 0.12	11.32 ± 2.08	10.64 ± 1.40	10.00 ± 2.91
nerol	0.10 ± 0.02	0.09 ± 0.03	0.12 ± 0.05	0.06 ± 0.05	0.10 ± 0.01	0.07 ± 0.06	0.09 ± 0.01	0.09 ± 0.03
Geraniol	7.69 ± 1.74	6.91 ± 2.32	1.93 ± 0.34	4.71 ± 4.15	0.11 ± 0.01	8.45 ± 1.19	7.63 ± 1.00	8.31 ± 0.29
exo-2-Hydroxy-1.8-cineole	0.03 ± 0.01	0.03 ± 0.01	0.38 ± 0.31	0.17 ± 0.24	0.36 ± 0.10	0.17 ± 0.24	0.03 ± 0.01	0.03 ± 0.01
**ALDEHYDES**								
Benzaldehyde	26.70 ± 6.06	24.01 ± 8.05	19.67 ± 2.76	22.18 ± 4.89	26.37 ± 0.28	25.91 ± 8.31	26.50 ± 3.48	29.03 ± 0.96
Hexanal	3.98 ± 0.90	3.58 ± 1.20	3.06 ± 0.16	3.99 ± 0.71	3.44 ± 0.84	5.25 ± 1.44	3.95 ± 0.52	3.71 ± 0.08
Furfural	99.07 ± 22.48	74.70 ± 6.55	74.45 ± 8.23	81.27 ± 19.69	97.53 ± 1.13	95.11 ± 32.46	98.32 ± 12.92	107.84 ± 3.57

*C, fermentation in grape must (control); Cu, fermentation in Cu-added grape must. Data are mean ± SD of three independent experiments. For each strain, the asterisk indicates significantly different values (one-way ANOVA, P < 0.05) between wines from control and Cu-added grape must*.

### Fermentations at pilot scale in cellar

Taking into account the potential ability of the strain MPR2-24 to remove copper content from wine, this strain was selected for pilot scale fermentation at cellar level in comparison to the commercial starter commonly used by the cellar (ES 454). The aim was to test the performance of this selected wild strain in real winemaking conditions. The tests were performed in must obtained by grapes collected in vineyard following the organic production system. Furthermore, the strain MPR2-24 was previously isolated during spontaneous fermentation of grapes collected in the same vineyard. The analysis of parameters correlated to a successful starter performance during fermentation, such as sugar consumption, ethanol production, reported in Table 5, showed that the wild strain possesses a fermentative performance comparable to the commercial starter. Also the content of some secondary compounds mainly involved in wine aroma, such as acetaldehyde and higher alcohols, detected in wine obtained by MPR2-24 was very similar to the level detected in wine produced by inoculating the commercial starter. The main differences between the two wines were related to the content of higher alcohols, mainly amyl alcohols, with higher content in wine obtained by wild strain than the level detected in wine fermented by commercial starter (344 and 293 mg L^−1^, respectively). In any case, it should be pointed out that the quantities of main by-products produced by the two starters respected the threshold values. Both indigenous and commercial starters showed a high strain implantation ability (92 and 100% for MPR2-24 and ES 454, respectively).

**Table 5:** Fermentation performance at cellar level by the selected indigenous *S. cerevisiae* strain in comparison to the commercial one.

**Parameters**	**Indigenous strain (MPR2-24)**	**Commercial strain (ES 454)**
Total acidity^a^	7,56	7,82
Volatile acidity^a^	0.18	0.22
Ethanol^b^	12.59	11.72
Acetaldehyde^c^	15,72	19,28
Ethylacetate^c^	63.25	64.61
*n*-Propanol^c^	33.77	46.63
Isobutanol^c^	31.03	48.86
Amyl alcohols^c^	344.17	292.98

a, g L^−1^;

b, % v/v;

c, *mg L^−1^*.

As regards the strain ability to reduce copper content in wine (CuRR), both the starters induced a reduction of this compound, although the wild strain possessed this capability at higher level than commercial starter (71 and 50%, respectively).

## Discussion

In consequence of the recent significant increase of organic wine sector, it is frequent to find grape must containing high level of copper residues, which is one of the most important biopesticides used in organic farms as copper formulates are effective against a high number of crops pests. High copper residual in grape must can be detrimental for the wine-making process and wine quality (Mira et al., 2007; Li et al., 2008). In fact, if the yeast strains performing the fermentative process are copper-sensitive, high amounts of this compound in must can inhibit yeast growth and activity.

The screening of copper tolerance among forty-seven *S. cerevisiae* strains was performed by the two different tests, growth on copper-added medium and fermentative activity in copper added grape must. The results obtained by the two different tests were comparable: the strains tolerating the highest copper concentration in YNB medium were the same which kept a good fermentative activity also in copper added must. These tests were very useful tools to identify very sensitive and tolerant strains and revealed the existence of high strain variability for this parameter, confirming previous data reporting that natural isolates of *S. cerevisiae* vary in their sensitivity to copper sulfate (Cavalieri et al., 2000; Mortimer, 2000). Some authors report that the analysis of traits of yeast population from specific area encompassed phenotypes that may reflect man-directed selection, for example copper resistance has been classified as a domestication trait (Warringer et al., 2011) and it may be an acquired adaptation as a result of the application of copper sulfate as a fungicide to treat powdery mildew in vineyards. These results support the idea that the isolation environment can exert a selective pressure on natural microflora. In our study, conversely, strain possessing copper tolerance at very different level, such as PP1-13 (very high copper tolerant) and MPR2-24 (very low copper tolerant) were isolated from fermented grapes collected in the same vineyard; the same findings were found for A20 and A13, both isolated from Aglianico del Vulture fermented grapes. These results suggest that, although some traits can be affected by natural selective pressure, it is necessary to consider the strain genetic basis for natural trait variation. The strain variability found was used to select four wild strains possessing this characteristic at the highest (PP1-13 and A20) and lowest level (MPR2-24 and A13). Looking at the evaluation of strain influence on copper content in fermentation, the four selected strains were firstly tested in SGM, a fermentation synthetic medium in which all the physical-chemical parameters can be standardized. The determination of copper content in final synthetic wines and yeast cells revealed that at the lowest copper residual in wine corresponded the highest content in yeast cells, indicating a strong strain ability to reduce the copper content in wine. This effect was inversely correlated with copper resistance: the most powerful strain in copper reduction was the most sensitive strain, MPR2-24. These results confirm the data previous reported by Sun et al. (2015), who demonstrated that “copper tolerance and copper adsorption ability of strains showed a negative correlation.” It's well known that a strict regulation of Cu homeostasis is required for *S. cerevisiae* cell survival and one of the mechanisms protecting cells from excess of copper is the reduction in copper uptake and its overload. Brady et al. (1994) found that the copper content in copper-tolerant yeast was lower than other strains when exposed to similar conditions, demonstrating that the mechanism for copper-resistance in *S. cerevisiae* was to reduce the intracellular uptake of copper (Wang and Chen, 2006). Adamo et al. (2012) hypothesize that one of the mechanisms of robustness toward copper might rely on hindering metal uptake. Some authors suggested a central role of the plasma membrane (Avery et al., 1996; Fernandes and Sa-Correia, 2001; Vagabov et al., 2008) and of the cell wall (Abbott et al., 2007) in the onset of tolerance to heavy metals. By our opinion, the high copper reduction ability of MPR2-24 strain might be most probably correlated to a biosorption mechanism. Factors affecting the metal biosorption in yeasts, such as status of biomass (living or non-living), types of biomaterials, properties of metal-solution chemistry, environmental conditions, were widely studied (reviewed in Wang and Chen, 2006), whereas studies reporting the influence of different *S. cerevisiae* strains on copper biosorption are very limited. Sun et al. (2015) reported that different *S. cerevisiae* strains are able to adsorb different quantity of copper during wine fermentation. These authors demonstrated that the main copper adsorption mechanism in *S. cerevisiae* during alcoholic fermentation was cell surface adsorption, as no copper was detected inside the yeast cells. It has been reported (Vinopal et al., 2007) that metallosorption capacity of the yeast wall is largely dependent on the outer mannoprotein layer. Park et al. (2003) reported that Cd^2+^ sorption capacity is proportional to thickness of the mannoprotein layer. The enzymatic removal of mannoproteins from the *S. cerevisiae* cell wall decreased the amount of sorbed Cd^2+^, Co^2+^, and Cu^2+^ (Brady et al., 1994). The enrichment of the *S. cerevisiae* cell wall with α-agglutinin derived mannoprotein enhanced the sorption capacity of genetically modified yeast for Cd^2+^ and Zn^2+^. Our results show that copper reduction was strain specific, with MPR2-24 strain exhibiting a very high ability to reduce copper content in wine, probably in consequence of high biosorption ability. We can speculate that this strain behavior can be correlated to a different cell wall composition of MPR2-24 in comparison to the other tested strains. In order to validate strain behavior in conditions that mimic wine fermentation, the strains were tested in NGM fermentation. The ability of MPR2-24 strain to reduce copper content in wine was confirmed also in fermentation of NGM. Other than the evaluation of strain ability to reduce copper content of natural wine, the aim of this trial was to evaluate the effect of copper addition on metabolic activity of copper sensitive and tolerant strains. As expected, copper affects the fermentative performance of sensitive strains; in particular, these strains started and completed the fermentative process later than copper tolerant strains, although all the fermentations were concluded with final very low residual sugars. The copper strain sensitivity affected wine volatile acidity; in fact copper sensitive strains in fermentation of copper-added must yielded wines with higher volatile acidity than wines obtained without copper. This result could be related to the stressful conditions suffered by sensitive strains in copper supplemented fermentation as an increase of volatile acidity after alcoholic fermentation is generally associated to a yeast stress signal (Bely et al., 2005; Cavazza et al., 2013). As regards the copper influence on strain metabolic activity, the determination of the secondary compounds affecting organoleptic quality of experimental wines showed that the production levels of these compounds were affected in sensitive strains, mainly in A13 (Tables 3, 4). Also this effect could be a consequence of mechanisms triggered as response of copper sensitive strains to metal stress. Since copper is a strong oxidizing agent (Adamo et al., 2012), the changing of metabolic activity of sensitive strains in copper added must fermentation can be a consequence of the reconfiguration of the glycolytic flux, a mechanism reported to regulate the response to oxidative stress in yeast cells and other eukaryotic organisms, such as human and plant (Morigasaki et al., 2008; Romano et al., 2015). Although the influence of copper on metabolic activity of sensitive strains, it has be underlined that all the secondary compounds detected in experimental wines were present at acceptable level (Swiegers et al., 2005).

On the basis of very interesting traits of MPR2-24 strain as biotechnological tool to reduce the copper content in wine, this wild strain was finally tested as starter culture in cellar pilot scale fermentation in comparison to a commercial starter. Also in real winemaking conditions this strain confirmed the traits exhibited during lab scale fermentation. Although MPR2-24 is a copper sensitive strain and the fermentations were performed in a grape must from organic vineyard, it completed successfully the fermentative process and showed high implantation ability, at a level comparable with fermentation performed by commercial starter culture. Therefore, this strain was able to survive and ferment in presence of copper, highlighting its good efficiency as starter culture. As reported by other authors, the choice of the right starter culture is crucial when there is a risk of high copper content in the grape must (Ferreira et al., 2006; Cavazza et al., 2013). The analysis of copper content in the two wines revealed that both the starters were able to reduce the copper content, although the indigenous starter at higher level than commercial one.

Our results showed that the study of copper adsorption in *S. cerevisiae* strains is an important tool to select starter strains able to conduct efficiently the fermentation process also in grape must containing too high copper residual. This situation is quite frequent in the last years, as a consequence of worldwide increase of organic wine sector, but it's well known that high copper residual in final wine, particularly existence with other heavy metals such as iron, manganese, zinc, nickel, lead, can cause some unaccountable risks for health consumers if metal concentrations are not kept under allowable limits (Naughton and Petróczi, 2008). Furthermore, copper content affects also wine quality since metallic ions have important role in oxide-reductive reactions resulting in wine browning, turbidity, cloudiness, and astringency. The wild strain MPR2-24, in addition to its ability to complete the fermentation and give acceptable flavor to the wine, possesses copper binding abilities and does therefore have great potential to be utilized as starter culture at industrial level. The use of this wild strain, that at the same time is able to perform successfully the alcoholic fermentation and reduce copper content in wine, represents an useful tool to assure not only the wine quality, but also to preserve the original color and flavor of wine.

Biotechnological reduction of copper content in wine is potentially a sustainable approach, as alternative to the chemical-physical methods, currently allowed by the official organizations, such as OIV. Continuing advances in yeast biology provide many opportunities for innovation and adaptation to a changing market. These will enable the development of new oenological practices based on the exploitation of new strains (Comitini et al., 2017). These new biotechnological tools can satisfy the increasing environmental pressures for a wine industry that is more efficient and more sustainable.

## Author contributions

AC contributed to the design of the work, to the acquisition and analysis of data, to draft the work and revising it, and ensured that questions related to the accuracy or integrity of any part of the work were appropriately investigated and resolved. RR contributed to the design of the work, to the management of experimental fermentation, to the statistical elaboration of data, to draft the work. LS contributed to the design of the work, to the chemical analysis of wine samples, to the interpretation of data for the work. GS contributed to the management of experimental fermentation, to the interpretation of data for the work. PR contributed to the design of the work, to the interpretation of data, to draft the work and revising it.

### Conflict of interest statement

The authors declare that the research was conducted in the absence of any commercial or financial relationships that could be construed as a potential conflict of interest.
