# Supplementary material for: Yeast Starter as a Biotechnological Tool for Reducing Copper Content in Wine
Source: Front Microbiol. 2018 Jan 10;8:2632. doi: 10.3389/fmicb.2017.02632 (PMC5767583; doi:10.3389/fmicb.2017.02632)
Supplement: Supplementary file 1 [file Table1.doc]

Table S1. Copper resistance, expressed as ratio between fermentative vigour in copper-added fermentations (Cu-FV) and the fermentative vigour without Cu addition (C-FV), of 47 *S. cerevisiae* strains.

| **Strain** | **C-FV** | **Cu-FV** | **Cu-FV/C-FV** |  |
| --- | --- | --- | --- | --- |
| MPR2 -24 | 0.10 | 0.03 | 0.35 |  |
| 10_2_ | 0.16 | 0.09 | 0.56 |  |
| AGME | 0.09 | 0.05 | 0.59 |  |
| 10_1_ | 0.15 | 0.09 | 0.62 |  |
| TA8-4SC2 | 0.15 | 0.09 | 0.62 |  |
| M3-80 | 0.10 | 0.06 | 0.63 |  |
| TA4-10 | 0.14 | 0.09 | 0.64 |  |
| SN41 | 0.14 | 0.09 | 0.68 |  |
| SC2-37 | 0.14 | 0.09 | 0.69 |  |
| A13 | 0.09 | 0.06 | 0.74 |  |
| SB5-18 | 0.11 | 0.09 | 0.79 |  |
| MPR2-18 | 0.10 | 0.08 | 0.86 |  |
| SB5-15 | 0.10 | 0.09 | 0.88 |  |
| MPR2- 26 | 0.10 | 0.09 | 0.90 |  |
| RB3-7SC2 | 0.10 | 0.09 | 0.91 |  |
| SA7-13 | 0.10 | 0.09 | 0.91 |  |
| PP1-1 | 0.10 | 0.09 | 0.91 |  |
| ND-14 | 0.10 | 0.09 | 0.91 |  |
| CB1-7SR3 | 0.09 | 0.09 | 0.93 |  |
| M1-47 | 0.09 | 0.09 | 0.93 |  |
| PP1-31 | 0.10 | 0.09 | 0.94 |  |
| MPR2- 43 | 0.10 | 0.09 | 0.94 |  |
| ND7 | 0.10 | 0.09 | 0.94 |  |
| PP1-15 | 0.10 | 0.09 | 0.94 |  |
| B7 | 0.09 | 0.09 | 0.95 |  |
| BP2-17 | 0.09 | 0.09 | 0.95 |  |
| M3-60 | 0.10 | 0.09 | 0.95 |  |
| MPR2-28 | 0.10 | 0.09 | 0.95 |  |
| 796 AWRI | 0.09 | 0.09 | 0.97 |  |
| MPR2-42 | 0.10 | 0.09 | 0.97 |  |
| PP2-22 | 0.10 | 0.10 | 0.97 |  |
| BP1-13 | 0.09 | 0.09 | 0.97 |  |
| BA-215 | 0.09 | 0.09 | 0.97 |  |
| BP1-33 | 0.09 | 0.09 | 0.97 |  |
| M3-59 | 0.10 | 0.09 | 0.97 |  |
| PP1-13 | 0.10 | 0.09 | 0.97 |  |
| CD2-6SC2 | 0.09 | 0.09 | 0.98 |  |
| FI5 | 0.09 | 0.09 | 0.99 |  |
| 4LB | 0.09 | 0.09 | 0.99 |  |
| A21 | 0.09 | 0.09 | 0.99 |  |
| BP1-29 | 0.09 | 0.09 | 0.99 |  |
| EC1118 | 0.09 | 0.09 | 1.01 |  |
| BP2-33 | 0.09 | 0.10 | 1.01 |  |
| B51 | 0.09 | 0.09 | 1.01 |  |
| A20 | 0.09 | 0.09 | 1.04 |  |
| A14 | 0.09 | 0.09 | 1.04 |  |
| 5TB8-60 | 0.09 | 0.09 | 1.04 |  |

**C-FV** = fermentative vigour expressed as gCO_2_/day measured at the third day of fermentation (control)

**Cu-FV** = fermentative vigour expressed as gCO_2_/day measured at the third day of fermentation (in Cu-added grape must)
